# Prediction of molecular subtypes from histology: AI-driven analysis of prostate cancer morphological patterns and therapeutic implications

**DOI:** 10.1038/s41698-026-01335-y

**Published:** 2026-03-19

**Authors:** Ramin Nateghi, Aoran Sun, Hao Dang, Nicole Handa, Marina Schnauss, Jamie Michael, Jae Woong Jang, Behtash G. Nezami, Madeline Saft, Clayton Neill, Sai Kumar, Edward M. Schaeffer, Hiten D. Patel, Ximing J. Yang, Lee A. D. Cooper, Ashley E. Ross

**Affiliations:** 1 Department of Urology, Northwestern University Feinberg School of Medicine, Chicago, IL USA; 2 Department of Pathology, Northwestern University Feinberg School of Medicine, Chicago, IL USA; 3 Department of Preventive Medicine, Northwestern University Feinberg School of Medicine, Chicago, IL USA; 4 Chan Zuckerberg Biohub, Chicago, IL USA

**Keywords:** Cancer, Computational biology and bioinformatics, Oncology, Urology

## Abstract

Molecular subtypes in prostate cancer significantly influence disease characteristics and treatment outcomes, yet obtaining this information requires specialized molecular testing. In this study, we develop and validate artificial intelligence models that can predict PAM50 and Prostate Subtyping Classifier (PSC) molecular classifications directly from standard hematoxylin and eosin (H&E)-stained biopsy slides. Using a cohort of 903 biopsy slides from 424 patients with matched molecular data, we demonstrate that our novel UNIv2-MIL framework, which fine-tunes a pre-trained pathology foundational model (UNIv2) using a multiple instance learning (MIL) strategy, achieves AUCs of 0.863 and 0.81 for PAM50 and PSC subtyping, respectively. Through computational clustering of high-attention regions, we identify histological patterns associated with molecular subtypes. Furthermore, in an independent validation cohort of 131 patients, we observed that patients with UNIv2-MIL predicted luminal subtypes tended to be more responsive to hormone therapy (HT) (*p* < 0.03). We also examined another independent cohort of 122 patients who transitioned from active surveillance to radical prostatectomy (RP) and observed that model-predicted PAM50 luminal B and PSC luminal proliferating scores showed significant correlation with adverse pathologic features (APFs) (*p* < 0.001 and *p* = 0.003, respectively). In conclusion, while further validation is needed, we have developed an AI-driven approach based on routine histopathology that has the potential to inform prostate cancer risk stratification and treatment response.

## Introduction

Prostate cancer is a significant global health challenge, with its impact varying across regions due to differences in healthcare access, screening, and treatment practices^1,2^. Recently, transcriptomics-based molecular subtyping of prostate cancer has shown the potential to individualize patient care, potentially improving outcomes^3–7^. The PAM50 basal (B), luminal A (LA), and luminal B (LB) subtypes, originally described in breast cancer, has demonstrated utility in categorizing both localized and metastatic castration-resistant prostate tumors, and may predict response to therapies targeting the androgen signaling axis^8–10^. The Prostate Subtyping Classifier (PSC), developed specifically with prostate cancer samples, classifies tumors into basal immune (BI), basal neuroendocrine (BN), luminal differentiated (LD), and luminal proliferating (LP)^11^. PSC has been shown in retrospective analysis to predict treatment responses to chemotherapy, radiotherapy, and hormonal interventions^12,13^. More specifically, research shows that patients with PAM50 luminal or PSC luminal tumors may be more responsive to androgen axis-based therapies than those with other subtypes^14,15^. In clinical practice today, PAM50 and PSC transcriptomic signature profiles can be identified on the Decipher gene expression assay. However, this requires tissue consumption, is not internationally accessible, and is associated with increased cost and processing time^16–19^.

In an effort to overcome these limitations, artificial intelligence and digital pathology have emerged as non-invasive alternatives for molecular subtype prediction directly from H&E-stained slides^20,21^. While the relationship between histomorphological features and transcriptomic profiles is not yet fully understood, machine learning algorithms can identify subtle pathological patterns that may correlate with gene expression signatures^22–24^. Research in this area is emerging but remains limited^20,25–27^. Most studies using AI for PAM50 classification focus on breast cancer, with only a few investigations exploring molecular subtype prediction in prostate cancer^28,29^.

Given the limited research in this promising area, this study introduces an innovative deep learning framework based on fine-tuning a pathology-trained foundational model to predict PAM50 and PSC signature subtypes from prostate biopsy specimens. Additionally, we explore correlations between model-identified pathological features and patient outcomes. We hypothesize that model-identified pathological features will be associated with response to androgen-axis-targeted therapies and adverse pathologic features.

## Results

### Study cohort characteristics

The primary cohort consisted of individuals diagnosed with prostate cancer having digitized pathology slides and available genomic data. In this cohort, 194 (45.0%) biopsies were molecularly classified as PAM50 LA subtype, 157 (36.4%) with LB subtype, and 80 (18.6%) with basal subtype. Stratified by PSC subtypes, the primary cohort was composed of 139 (32.3%) biopsies with LD, 95 (22%) with LP, 188 (43.6%) with BI, and 9 (2.1%) with BN (Supplementary Fig. 1). Clinical and pathologic characteristics of the primary cohort stratified by PAM50 and PSC molecular subtypes are shown in Supplementary Tables 1, 2, respectively.

Our histopathology-based molecular subtype classifiers were evaluated for their relationship to clinical outcomes on two additional independent cohorts. Our second study cohort was comprised of 131 patients with available hormonal therapy treatment data and digitized pathology slides, of whom 112 (85%) demonstrated HT responsiveness while 19 (15%) were classified as HT non-responsive (clinical and pathological characteristics listed in Supplementary Table 3). Using the third cohort, we evaluated the model’s ability to predict adverse pathologic features in 122 patients transitioning from active surveillance to radical prostatectomy treatment. Of the 122 active surveillance patients who subsequently underwent radical prostatectomy, 39 (32%) exhibited APFs, and 83 (68%) did not (clinical and pathologic features listed in Supplementary Table 4).

### AI-driven PAM50 and PSC subtype prediction performance

**Fig. 1:**
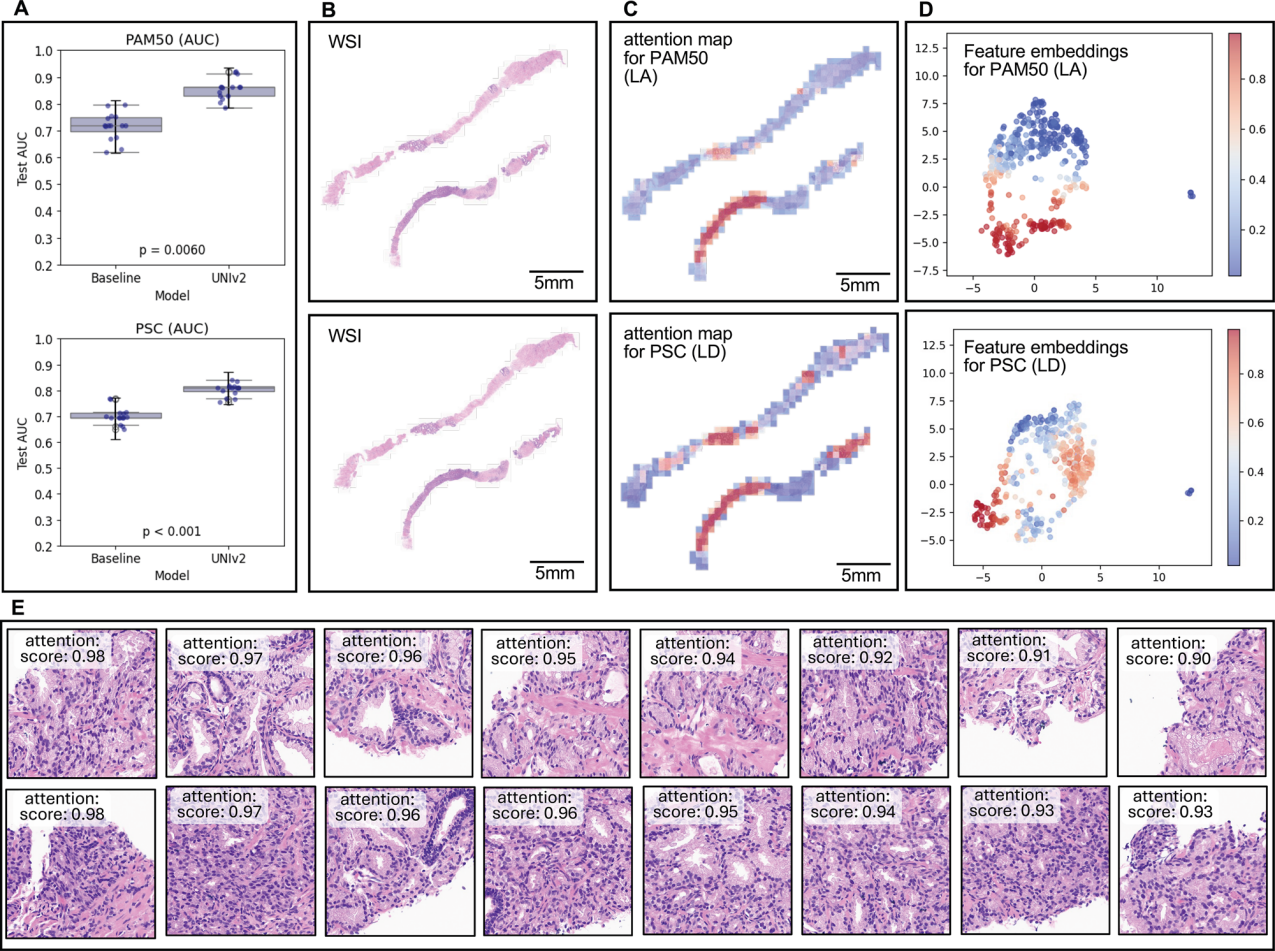
Classification performance and interpretability of the UNIv2-MIL model for PAM50 and PSC subtyping. **A** Comparison of AUC between the baseline and UNIv2-MIL models, with UNIv2-MIL outperforming the baseline in both PAM50 and PSC subtyping. **B**, **C** Attention maps overlaid on representative H&E biopsy slide, highlighting regions significant for PAM50 and PSC subtyping. **D** UMAP embeddings of features extracted from slide tiles, color-coded by attention weight. **E** Top eight most informative tiles, ranked by attention weight, for PAM50 (upper row) and PSC (bottom row) subtyping.

In the testing set, both the PAM50 and PSC classification performance metrics were improved compared to our baseline model with a novel finetuned UNIv2-MIL approach (Fig. 1A). For PAM50 subtyping, the UNIv2-MIL model showed statistically significant

performance improvements over the baseline model, achieving a substantially higher AUC of 0.863 (95% CI: 0.78–0.93) compared to the baseline model’s AUC of 0.718 (95% CI: 0.61–0.81), and an improved F1-score of 0.727 versus 0.576 (*p*-value = 0.006). For PSC subtype classification, UNIv2-MIL outperformed the baseline, achieving an AUC of 0.81 (95% CI: 0.74–0.87) compared to 0.694 (95% CI: 0.62–0.77), with corresponding F1-scores of 0.55 and 0.44 (*p*-value < 0.001), respectively. Figure 1B, C demonstrate the visual interpretability of our UNIv2-MIL model’s attention maps for a representative correctly classified H&E biopsy slide from a patient with PAM50 LA and PSC LD subtype (additional slides shown in Supplementary Fig. 2). The overlaid attention maps highlight regions identified by the model as highly significant for PAM50 LA and PSC LD subtyping, showing some degree of overlap in areas used to predict both subtypes. The intensity of the heatmap corresponds to the attention weight assigned by the model, with regions highlighted in red indicating informative for PAM50 LA and PSC LD subtypes, and blue representing areas of lower importance. These panels provide insight into which histological regions the model prioritizes for subtype classification, helping to link model predictions to interpretable tissue features.

Across all testing biopsy blocks, we quantified the spatial overlap between all pairs of PAM50 and PSC subtypes via Pearson correlation of the raw attention scores generated by the models. The strongest spatial correlation was observed between PSC basal immune and PAM50 basal (*r* = 0.58, *p* = 0.034), followed by moderate correlation between PSC luminal proliferating and PAM50 luminal B (*r* = 0.25, *p* = 0.057), while the most pronounced negative spatial correlation occurred between PSC luminal proliferating and PAM50 basal (*r* = −0.70, *p* = 0.014). Other subtype pairs did not exhibit statistically significant correlations. Figure 1D illustrates the UMAP embeddings of features extracted from patches of the presented slide, with colors aligning with the color theme used to overlay attention weights on the slide. The UMAP plots show that the patches receiving high attention for PAM50 LA and PSC LD occupy approximately the same region in feature space, confirming the overlap in informative tissue regions identified by the model. Figure 1E visualizes the top eight most informative tiles, with the highest attention weights assigned by the model, where the upper row corresponds to PAM50 subtyping and the lower row to PSC subtyping. Notably, attention is not restricted to high-cellularity tumor regions, and infiltrative, less differentiated, and stroma-rich regions are also recognized by the model as informative for subtype classification, with PAM50 LA tiles generally showing well-formed glands and larger intervening stromal areas, and PSC LD tiles including regions with higher cellularity.

### Relationship between histological patterns and molecular subtypes

**Fig. 2:**
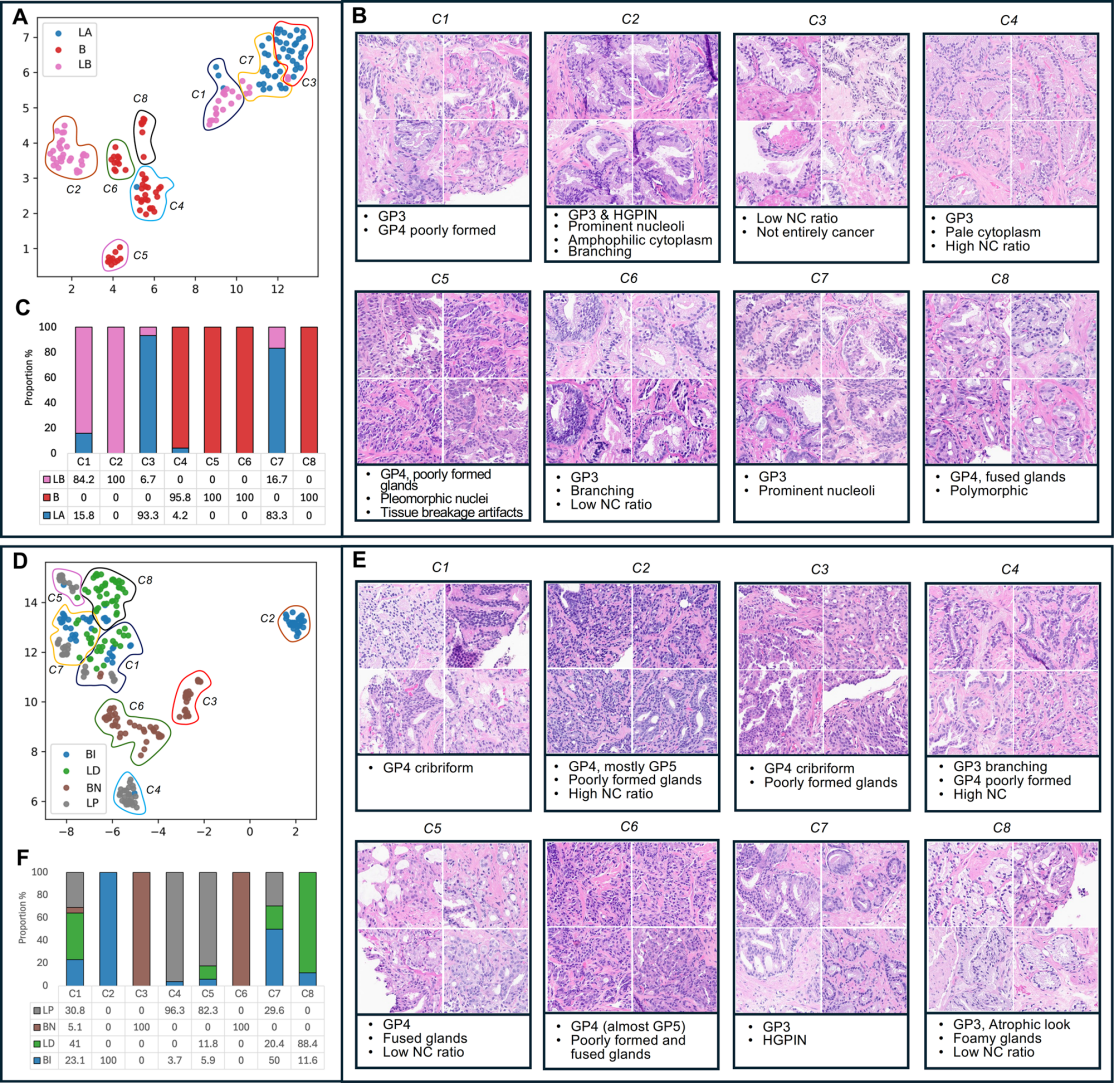
Correlation between morphological patterns and molecular subtypes in prostate cancer. UMAP projections of high-attention image tiles from PAM50 (**A**) and PSC (**D**) subtyping models, color-coded by molecular subtypes. K-means clustering (k = 8) was applied, with boundaries overlaid. Representative tiles from each cluster (C1–C8) for PAM50 (**B**) and PSC (**E**), with pathologist interpretations summarized below each cluster. Distribution of imaging clusters across PAM50 (**C**) and PSC (**F**) subtypes.

To investigate the correlations between histological morphologies in pathology slides with molecular subtypes, a computational analysis was performed on high-attention tile features derived from the testing cohort using PAM50 and PSC classification models. To ensure that only the most informative tiles were considered, tiles receiving attention scores higher than 0.8 were selected. This threshold was chosen to ensure that enough tiles from all subtypes were included in the analysis, while avoiding excessively strict cutoffs that could omit relevant regions, and looser thresholds that might include less informative areas. Figure 2A, D presents UMAP projections of the learned latent feature representations derived from image tiles receiving the highest attention scores by our PAM50 and PSC subtyping models, respectively, where each dot represents one tile with the color representing the molecular subtypes (LA, LB, B for PAM50 and LD, BI, LP, and BN for PSC) assigned by the models. To further characterize these patterns, we applied K-means clustering to group the informative tiles into eight distinct clusters for both PAM50 and PSC subtypes, chosen as a balance between capturing sufficient histologic variation and maintaining interpretability, as delineated by the boundaries in Fig. 2A, D. Representative tiles from each cluster are displayed in Fig. 2B, E, respectively. These tiles were identified by the model through its attention mechanism during subtyping. A genitourinary pathologist reviewed these model-identified tiles within the clusters to qualitatively identify distinct pathological patterns associated with each cluster, summarized under each representative tiles in panels B and E. The distribution of PAM50 subtypes (LA: luminal A, LB: luminal B, B: basal) showed distinct patterns across the eight clusters (C1–C8), see Fig. 2C. Clusters C5, C6, and C8 contained exclusively basal subtype (100%), with these clusters exhibiting more aggressive pathological features, containing Gleason pattern (GP) 4, with poorly formed glands, and pleomorphic nuclei. Clusters C3 and C7 were predominantly luminal A (93.3% and 83.3%, respectively), characterized by a less aggressive disease with GP 3, and a low nuclear-to-cytoplasmic (NC) ratio. The luminal B subtype was concentrated in cluster C2 and partially in C1 (100%, 84.2%, respectively), with C2 exhibiting GP3 and high-grade prostatic intraepithelial neoplasia (HGPIN) features with prominent nucleoli and amphophilic cytoplasm, and C1 with less aggressive, poorly formed GP4. The proportions exhibited a strong correlation between PAM50 molecular subtypes and specific histopathological features. For example, basal-type tumors are mostly associated with higher-grade, more aggressive characteristics, luminal A with less aggressive features, and luminal B with distinct morphological patterns, including amphophilic cytoplasm and prominent nucleoli, and poorly formed GP 4.

We further investigated the distribution of image pattern clusters (C1–C8) identified by the PSC subtyping model across four PSC subtypes (BI, BN, LD, and LP), see Fig. 2F. Clusters C3 and C6 consisted exclusively of BN subtype (100%), with C3 exhibiting GP 4 cribriform architecture with poorly formed glands and C6 showing more aggressive GP 5 with poorly formed and fused glands. Cluster C2 contained only the BI subtype (100%), characterized by GP 4/GP 5 patterns with poorly formed glands, and a high nuclear-to-cytoplasmic ratio. Clusters C4 and C5 were both heavily dominated by LP subtype (96.3% and 82.3%, respectively), though C5 showed more heterogeneity with minor contributions from BI (11.8%). Histologically, C4 presented with GP 3 branching and GP 4 poorly formed structures with a high NC ratio, while C5 exhibited GP 4 with fused glands but a low NC ratio. Cluster C8 primarily represented the LD subtype (88.4%), associated with GP3, atrophic-appearing cancer, foamy glands, and a low NC ratio. Cluster C7 showed a more balanced distribution characterized by less aggressive GP 3 and HGPIN features. These findings reveal important biological implications across the PSC subtypes. BN subtypes are strongly linked to aggressive histology, including cribriform and poorly formed glands typical of higher Gleason patterns (GP 4-5), suggesting their role in advanced disease. LP subtypes display various morphological patterns, mostly associated with intermediate to high GP 4 disease. The BI subtype demonstrates distinctive high-grade features (GP4/GP5) with an elevated nuclear-to-cytoplasmic ratio, suggesting active cellular processes potentially linked to immune infiltration within the tumor microenvironment. These findings provide additional pathological insights into the biological characteristics of prostate cancer that may help guide interpretation from standard pathology slides. Supplementary Fig. 3 illustrates the pairwise correlation matrix calculated by the cosine similarity index between the clusters identified by the PAM50 and PSC classification models. The generally low correlation values between clusters suggest that each cluster captures distinct pathological patterns in prostate cancer histopathology.

### Association between predicted molecular subtype scores and treatment outcomes

To examine potential associations between the model’s molecular subtype predictions and patient outcomes, we analyzed the model-predicted PAM50 and PSC scores with respect to treatment outcomes. As shown in Table 1, we observed significant associations between certain predicted molecular subtype scores predicted from biopsy histology and the outcome. Notably, luminal B scores predicted by the PAM50 model were significantly higher in HT-responsive patients compared to non-responsive patients (median [IQR] 0.21 [0.07, 0.39] vs 0.05 [0.00, 0.16], *p* = 0.007). Point biserial correlation analysis further confirmed these findings, with luminal B scores showing a significant positive correlation (*r* = 0.2196, *p* = 0.0117) with HT responsiveness. Similarly, model-predicted luminal proliferating subtype scores, which shares some similarities with luminal B in terms of biological features, particularly regarding high androgen receptor (AR) activity^9,12^, were also significantly higher in HT-responsive patients compared to non-responsive patients (median [IQR] 0.09 [0.04, 0.17] vs 0.05 [0.01, 0.10], *p* = 0.026), with point biserial correlation analysis showing a positive correlation (*r* = 0.1495, *p* = 0.0883). These findings suggest that tumors with elevated luminal B and luminal proliferating characteristics are more likely to respond favorably to hormone therapy.

**Table 1:** Association between predicted molecular subtype scores and prognostic HT outcomes

Model scores	HT non-responsive *N* = 19^a^	HT responsive *N* = 112^a^	Overall *N* = 131^a^	*p*-value^b^	Correlation^c^	*p*-value^c^
Luminal A	0.44 (0.13, 0.61)	0.30 (0.08, 0.55)	0.32 (0.12, 0.56)	0.14	-0.1264	0.1502
Luminal B	0.05 (0.00, 0.16)	0.21 (0.07, 0.39)	0.17 (0.05, 0.36)	**0.007**	**0.2196**	**0.0117**
Basal	0.00 (0.00, 0.06)	0.00 (0.00, 0.00)	0.00 (0.00, 0.00)	0.3	-0.0598	0.4978
Luminal differentiated	0.31 (0.26, 0.47)	0.32 (0.18, 0.46)	0.31 (0.21, 0.46)	0.7	-0.01948	0.8252
Luminal proliferating	0.05 (0.01, 0.10)	0.09 (0.04, 0.17)	0.09 (0.03, 0.16)	**0.026**	0.1495	0.0883
Basal immune	0.60 (0.43, 0.69)	0.49 (0.38, 0.65)	0.51 (0.39, 0.65)	0.2	-0.0949	0.2810
Basal neuroendocrine	0.03 (0.02, 0.04)	0.03 (0.02, 0.04)	0.03 (0.02, 0.04)	>0.9	0.00211	0.9809

^a^Median (Q1, Q3); *n* (%).

^b^Wilcoxon rank sum test.

^c^Point biserial.

Bold values indicate statistically significant associations (*p*-value <0.05).

**Fig. 3:**
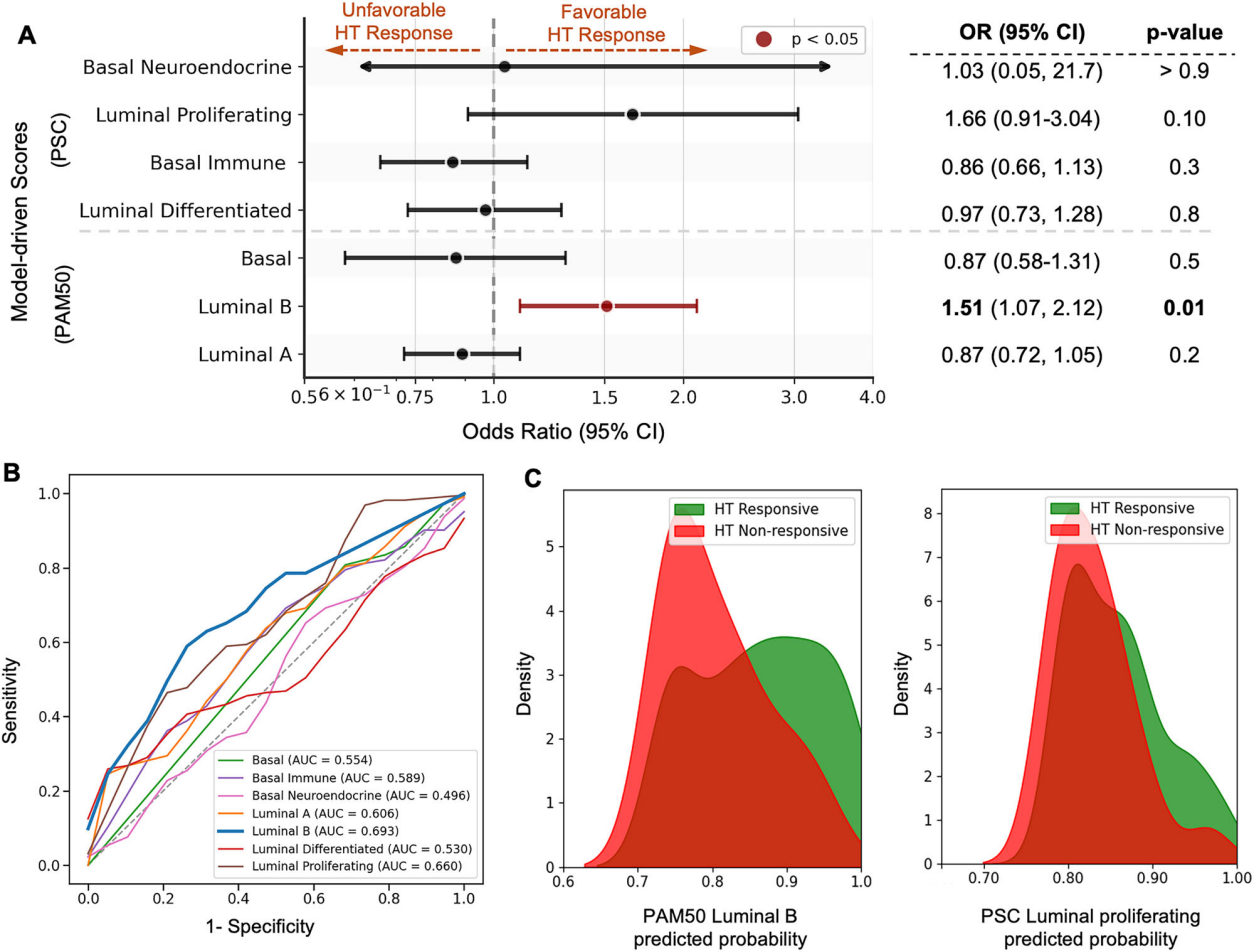
Association of predicted molecular subtype scores with hormone therapy response. **A** Forest plot showing odds ratios (ORs) and 95% confidence intervals (CIs) for each predicted molecular subtype score in predicting HT response. The PAM50 Luminal B subtype was significantly associated with a favorable HT response (*p* = 0.01). **B** ROC curves for each subtype, with PAM50 Luminal B achieving the highest area under the curve (AUC = 0.693). **C** Kernel density estimates (KDE) of predicted probabilities for PAM50 Luminal B (left) and PSC Luminal Proliferating (right) scores, stratified by HT response status, showing greater separation between responsive and non-responsive patients for the Luminal B subtype.

To further validate the predictive utility of these molecular subtype scores predicted by the models from digitized biopsies, we performed univariate logistic regression analysis to assess associations with hormone therapy (HT) responsiveness. Among all molecular

subtypes scores examined, luminal B subtype demonstrated a statistically significant association with hormonal therapy responsiveness (OR = 1.51, 95% CI: 1.07–2.12, *p* = 0.01), indicating that luminal B-like tumors are associated with increased likelihood of favorable response to hormonal therapy (Fig. 3A). All other predicted PAM50 and PSC molecular subtype scores showed no significant associations with treatment outcome (all *p* > 0.05) (Fig. 3A). Receiver operating characteristic (ROC) curve analysis also demonstrated the discriminative performance of each score for predicting HT response, with luminal B achieving the highest predictive accuracy (AUC = 0.693), followed by luminal proliferating (AUC = 0.660) (Fig. 3B). Density plots further illustrated the distribution of scaled predicted scores using kernel density estimates (KDE), showing clear separation between HT responsive and non-responsive patients for both PAM50 luminal B and PSC luminal proliferating scores (Fig. 3C), supporting the predictive value of these features, particularly the Luminal B subtype. On multivariable logistic regression adjusting for clinical covariates, including maximum GP4 percentage and maximum core-level cancer percentage (all per 1%-unit increment), luminal B (OR = 1.33, 95% CI: 0.94–1.90, *p* = 0.11), and luminal proliferating scores (OR = 1.32, 95% CI: 0.74–2.36, *p* = 0.3) showed a non-significant trend toward increased odds of HT responsiveness, while GP4 was not significant (OR = 1.00, 95% CI: 0.99–1.02, *p* = 0.8).

The threshold used to filter low-confidence scores before aggregation was set at 0.5 throughout this analysis; however, we further investigated the robustness of these findings using an alternative threshold of 0.3. Similarly, the newly aggregated PAM50 luminal B and PSC LP scores remained significantly associated with HT responsiveness (LB: median [IQR] 0.22 [0.08, 0.42] vs 0.08 [0.00, 0.17], *p* = 0.002, and LP: median [IQR] 0.10 [0.05, 0.19] vs 0.07 [0.01, 0.13], *p* = 0.036) (Supplementary Table 5). More extreme or looser thresholds may either exclude diagnostically relevant regions or introduce noise from low-confidence predictions.

### Relationship between predicted molecular scores and adverse pathologic features

To investigate correlations between molecular subtypes and adverse pathologic features at RP, we used the third cohort where PAM50 and PSC molecular subtype scores were predicted from the final digitized biopsy specimen preceding surgery. Predicted molecular scores below 0.5 were set to zero, while higher values were retained, then aggregated across various cores taken from the same biopsy to generate patient-level scores. These scores were then analyzed for associations with APFs (Table 2). Patients

**Table 2:** Association between predicted molecular subtype scores and adverse pathologic features at RP

Model scores	Non-APFs *N* = 83^a^	APFs *N* = 39^a^	Overall *N* = 122^a^	*p*-value^b^	Correlation^c^	*p*-value^c^
Luminal A	0.46 (0.29–0.76)	0.30 (0.44–0.75)	0.42 (0.34, 0.76)	0.36	0.09	0.32
Luminal B	0.00 (0.00–0.00)	0.01 (0.00–0.11)	0.00 (0.00, 0.00)	**<0.001**	0.325	**<0.001**
Basal	0.00 (0.00–0.01)	0.00 (0.00–0.01)	0.00 (0.00, 0.00)	0.26	-0.11	0.2
Luminal differentiated	0.31 (0.29–0.60)	0.21 (0.38–0.59)	0.27 (0.33, 0.61)	0.49	0.043	0.63
Luminal proliferating	0.00 (0.00, 0.00)	0.07 (0.00–0.07)	0.03 (0.00, 0.03)	**0.003**	0.239	**<0.001**
Basal immune	0.35 (0.257–0.61)	0.26 (0.25–0.51)	0.32 (0.25, 0.58)	0.18	-0.118	0.23
Basal neuroendocrine	0.0 (0.00, 0.00)	0.00 (0.00, 0.00)	0.00 (0.00, 0.00)	1	–	**–**

^a^Median (Q1, Q3); *n* (%).

^b^Wilcoxon rank sum test.

^c^Point biserial.

Bold values indicate statistically significant associations (*p*-value <0.05).

with APFs exhibited higher luminal B scores relative to those with favorable outcomes (median [IQR] 0.01 [0.00–0.11] vs 0.00 [0.00–0.00], *p* < 0.001), with point biserial correlation substantiating a robust positive relationship (*r* = 0.325, *p* < 0.001). Likewise, luminal proliferating signatures showed meaningful association with APFs (median [IQR] 0.07 [0.00–0.07] vs 0.00 [0.00–0.00], *p* = 0.003) and demonstrated considerable positive correlation (*r* = 0.239, *p* < 0.001). On univariable logistic regression analysis, several clinical variables showed significant associations with APFs. Biopsy Gleason grade prior to RP (OR = 4.43, 95% CI: 1.48–13.3, *p* = 0.008) and GP4 percentage on biopsy prior to RP (OR = 28.1, 95% CI: 4.15–190, *p* < 0.001) were significantly associated with APFs. Age, BMI, PSA, race, ethnicity, and other clinical variables showed no significant associations with APFs (all *p* > 0.05). Among model-predicted molecular subtype scores, luminal B scores were strongly associated with APFs (OR = 2.45, 95% CI: 1.40–4.30, *p* < 0.001), as were luminal proliferating scores (OR = 2.58, 95% CI: 1.16–5.73, *p* = 0.010). Other PAM50 and PSC subtype scores did not show significant associations with APFs. On multivariable logistic regression adjusting for maximum GP4 and core-level cancer percentage (per 1% unit increase), luminal B scores showed a non-significant trend toward an association with APFs (OR = 1.69, 95% CI: 0.88–3.26, *p* = 0.12), whereas luminal proliferating scores remained significant and demonstrated the highest odds among all variables (OR = 2.41, 95% CI: 0.99–5.84, *p* = 0.052). GP4 percentage also remained an independent predictor of APFs (OR = 1.29, 95% CI: 1.11–1.50, *p* < 0.001). These findings suggest that prostate cancers with luminal B and luminal proliferating molecular phenotypes are more likely to demonstrate adverse pathologic features at radical prostatectomy, potentially reflecting their heightened proliferative capacity.

**Fig. 4:**
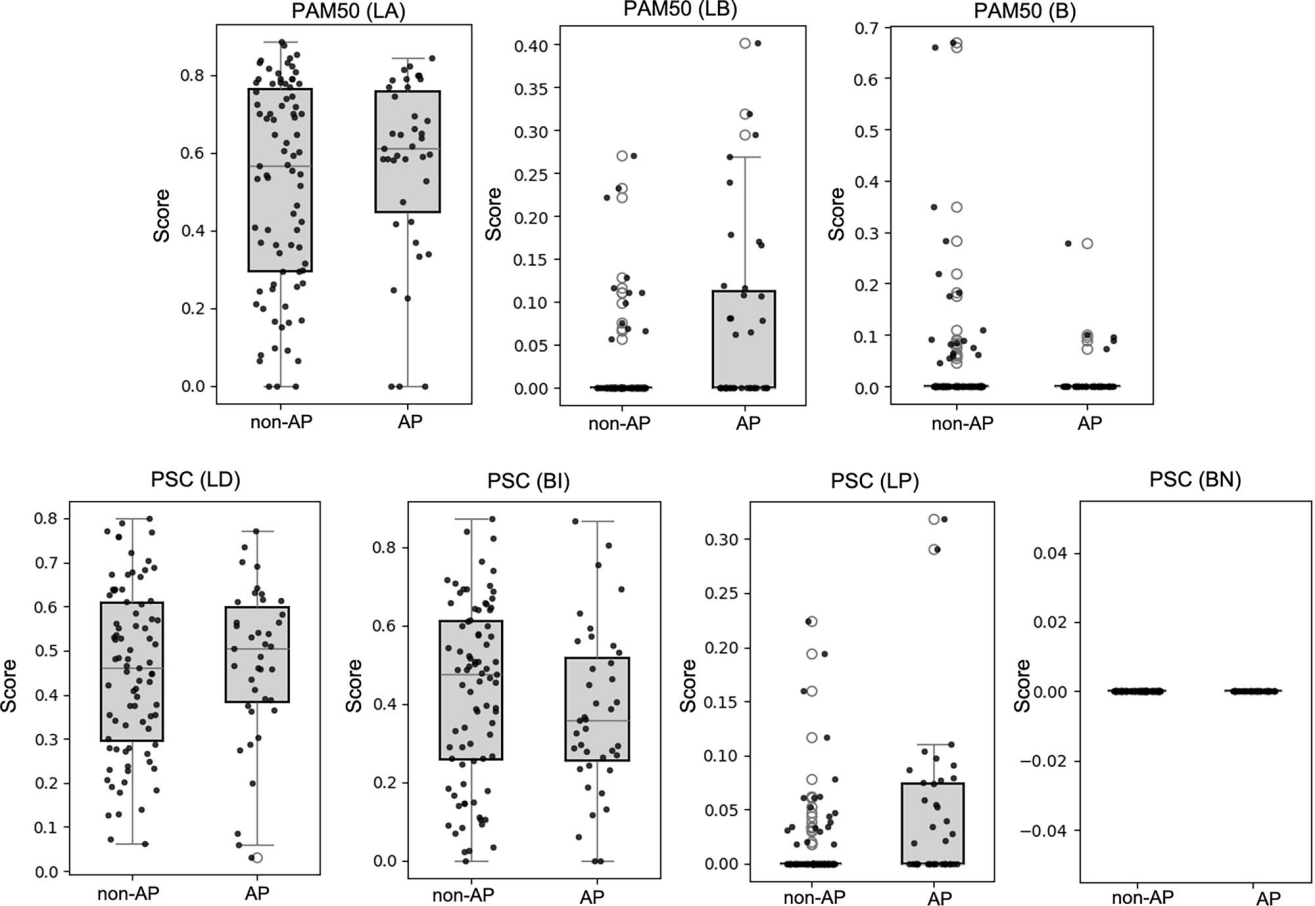
Patient-level molecular subtype scores stratified by adverse pathologic features. Box plots comparing PAM50 (LA, LB, B) and PSC (LD, BI, LP, BN) subtype scores between patients with APFs and those without at radical prostatectomy. Boxes represent interquartile range, horizontal lines indicate medians, and points represent individual patients. Patients with adverse features showed higher LB and LP scores.

To further contextualize these findings beyond statistical significance, we additionally plotted patient-level distributions of the aggregated molecular subtype scores. As shown in Fig. 4, patients with adverse pathologic features demonstrated notably elevated luminal B scores compared to those with favorable outcomes, with a visible shift in the distribution toward higher values. Similarly, luminal proliferating scores showed a modest but discernible increase in the adverse pathology group.

## Discussion

Molecular assays have increasingly emerged as tools for prognostic and predictive testing in prostate cancer. However, these tests are costly, resource-intensive, and often have long turnaround times, which can limit their routine use in clinical settings. AI-powered digital pathology offers a potential approach to support predictions of tumor biology directly from routine histopathology samples. In this study, we present a deep learning framework that classifies PAM50 and PSC molecular subtypes of prostate cancer using standard H&E-stained tissue. By analyzing these slides computationally, we observed relationships between tissue morphology and molecular classifications, although further validation on an expanded cohort is needed before clinical adaptation.

Central to our work is the training strategy we devised for adapting foundational pathology models to molecular subtype prediction. In typical digital pathology pipelines for classification tasks, a pretrained feature encoder, whether a general vision network or a pathology-specific foundational model, is often frozen, and only a downstream aggregation model (e.g., MIL or transformer head) is trained. While this setup reduces computational costs, it limits the feature encoder’s ability to adapt to the specific downstream task. In contrast, we explored two distinct approaches: (1) in our baseline model, we used an end-to-end trainable architecture that jointly optimizes both the CNN encoder and the attention-based MIL aggregator using a randomly sampled subset of tiles, and (2) in our advanced model, we developed a two-stage fine-tuning framework that adapts UNIv2 foundational pathology representations for improved subtype-specific feature learning and classification. The second methodology led to markedly improved results, achieving an AUC of 0.863 (compared to our baseline of 0.81). This highlights the benefits of fine-tuning foundational models for subtype-specific adaptation. One limitation of our data generation approach is that the genomic subtypes derived from a single Decipher-tested biopsy block, which were used as ground truth in our digital pathology pipeline, were applied to all tumor-containing blocks from the same case. This assignment may encompass regions that are less directly representative of the underlying molecular profile, attenuating observed associations between histological and genomic features. Nevertheless, the tile-selection mechanisms implemented in our model facilitate learning from the most informative regions for molecular subtyping, which may reduce the impact of this limitation.

From a pathological standpoint, our computational analysis of histological patterns suggested that basal-like tumors tend to exhibit more aggressive morphological features, whereas luminal-like cancers show varying degrees of differentiation that correspond to their biological characteristics. These observations provide visual cues that could aid in recognizing molecular subtypes during routine examination; however, further validation on an expanded cohort is required to confirm the generalizability of these associations. We also observed that certain histological patterns overlapped between PAM50 and PSC subtypes. Based on our genitourinary pathologist’s review of high-attention tiles, basal-like PAM50 tumors and BN PSC tumors shared features such as poorly formed or fused glands with higher Gleason patterns, luminal B PAM50 and LP PSC tumors tended to exhibit intermediate features including prominent nucleoli and partially poorly formed glands, and luminal A PAM50 and LD PSC tumors showed low-grade, well-differentiated glandular structures with low nuclear-to-cytoplasmic ratio. These findings align with previous research by Zhao et al.^9^ and Weiner et al.^12^, who reported that basal subtypes, particularly BN, were associated with higher Gleason scores and increased proliferation. These overlaps are based on visual examinations, but we believe that the pathological differences between subtypes may sometimes be subtle.

From a clinical and therapeutic perspective, previous research has established some connections between molecular subtypes and response to hormonal therapies. One study by Zhao et al.^9^ demonstrated that patients with PAM50 luminal B tumors and PSC LP subtypes generally show improved response to hormonal therapy compared to those with basal subtypes. The biological basis for these differential responses has been attributed to varying degrees of androgen receptor signaling dependency across molecular subtypes. Recent results from the NRG GU006 (BALANCE) trial further support the clinical relevance of PAM50 luminal B subtypes, showing that patients with these tumors benefit from the addition of apalutamide to salvage radiotherapy^30^. Motivated by this, we further assessed the clinical relevance of AI-derived molecular subtypes by examining their associations with therapeutic response and adverse pathologic features. Notably, higher model-predicted scores for luminal B and luminal proliferating subtypes were significantly associated with favorable response to hormone therapy, supporting earlier findings by McKenney et al.^31^. Among these, the luminal B subtype demonstrated the strongest predictive performance, with a statistically significant association in logistic regression (OR = 1.51, 95% CI: 1.07–2.12, *p* = 0.01) and the highest discriminative accuracy (AUC = 0.693). In addition, we observed that these same subtypes were significantly enriched in patients with adverse pathologic features. Their elevated scores and moderate-to-strong correlations with APFs suggest that, despite increased hormone sensitivity, these tumors may exhibit a more aggressive biological phenotype, marked by enhanced proliferative and invasive potential.

Our study has several limitations. The limited size and imbalanced distribution of molecular subtypes in our cohort, particularly the underrepresentation of rare subtypes like basal neuroendocrine, limit the robustness of our findings. However, this distribution reflects real-world incidence of the different subtypes, with studies by Zhao et al.^9^ and Feng et al.^32^ reporting that luminal subtypes account for ~62–67% of prostate cancer cases. Our second and third cohorts were relatively small, especially the second cohort, which included few hormonal therapy non-responder patients, limiting the evaluations. The relatively small sample size also restricted our ability to perform more comprehensive multivariable analyses. Nevertheless, the preliminary results are promising, suggesting further validation in larger prospectively collected cohorts. Future research should prioritize multi-institutional prospective studies with expanded sample sizes to enable robust multivariable assessments, including evaluation of the model’s performance across high- and low-volume metastatic disease and other clinical variables. Integrating these findings with additional clinical, imaging, and molecular data could also deepen our understanding of the biological mechanisms underlying observed associations and improve predictive performance. Additionally, studying how histological-molecular correlations evolve during disease progression could reveal dynamic biomarkers for adaptive therapeutic strategies.

In conclusion, we successfully developed and validated a novel deep learning model that predicts molecular subtypes and provides insights into treatment response in prostate cancer directly from standard H&E slides. Given the growing accessibility of digital pathology, this AI-based approach can offer additional insights into molecular subtypes and complement routine pathology workflows to support prognostic assessments. With our findings providing initial evidence, further validation in larger and more diverse cohorts remains necessary before clinical implementation. These results represent an initial step toward more accessible and timely molecular assessment using digital pathology slides in prostate cancer management.

## Methods

### Study cohorts

**Fig. 5:**
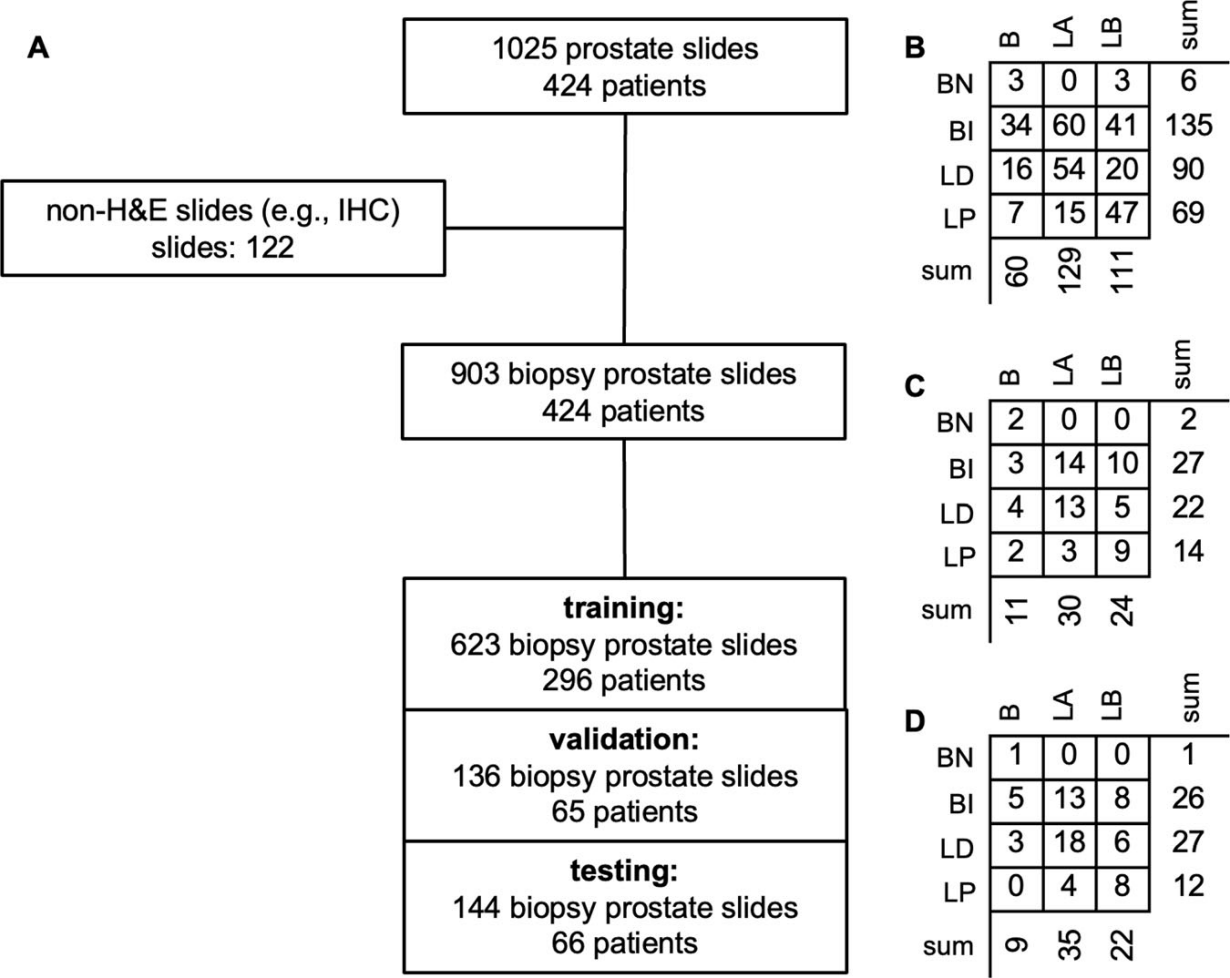
Patient selection strategy and distribution of samples in the first cohort. **A** A cohort comprising 903 biopsy slides from 424 prostate cancer patients, stratified at the patient level into training, validation, and testing datasets. **B**–**D** Distribution of biopsy blocks across training, validation, and testing datasets according to PAM50 and PSC molecular subtypes, showing the representation of each subtype within each dataset.

We utilized three distinct cohorts: a primary cohort comprising 903 H&E-stained biopsy slides from 424 patients diagnosed with prostate cancer that also underwent molecular decipher testing, an independent cohort of 2432 H&E-stained biopsy slides from 131 patients treated either with ADT monotherapy or doublet therapy including ADT plus an androgen receptor pathway inhibitor (ARPI) (i.e. an androgen receptor blocker or cyp17 inhibitor), and a cohort of 2267 H&E-stained biopsy slides from 122 patients who were initially on active surveillance and opted to receive radical prostatectomy (RP). In the second cohort, we refer to both monotherapy and doublet ADT groups collectively as patients receiving hormonal therapy (HT). The primary cohort served as the foundation for developing and validating our PAM50 and PSC subtyping models, with slides linked to the Decipher Genomic Resource Information Database (GRID) to obtain molecular PAM50 and PSC subtyping data derived from Decipher testing, which is based on transcriptomic profiling of the underlying biopsy specimens done by Veracyte, Inc San Diego, CA, using whole transcriptome analysis. At our institution, the biopsy block with the highest grade and tumor volume is selected, and the entire block is submitted for Decipher testing to obtain PAM50 and PSC molecular subtypes. In a case where the highest-grade block is not the highest volume block, the RNA extraction is performed on the highest-grade block. The PAM50 system classifies tumors into three subtypes of B, LA, and LB, while the PSC stratifies into four categories of BI, BN, LD, and LP^9,12^. To ensure precise integration, after excluding non-H&E slides, biopsy slides from the primary cohort were mapped to molecular subtypes at the biopsy block level using patient and biopsy specimen identifiers. This mapping ensured that all digitized blocks originating from a biopsy from which a block was used for Decipher testing were assigned a single, definitive PAM50 and PSC subtypes derived from the Decipher-tested block. These subtypes served as the ground-truth labels for model training and evaluation (Supplementary Fig. 4). The cohort was then stratified at the patient level with 70% for training, 15% for validation, and 15% for testing (Fig. 5A). The distribution of biopsy blocks, categorized by PAM50 (B, LA, LB) and PSC (BI, BN, LD, LP) subtypes, is depicted in Fig. 5B–D for the training, validation, and testing datasets. The second independent cohort was employed to evaluate whether model-derived molecular subtype scores could predict therapeutic response to HT. All slides were obtained from standard needle biopsy samples and scanned at Northwestern Memorial Hospital (NMH) using a Leica Systems Aperio GT 450 scanner at 40× magnification. Biopsies were from patients diagnosed with prostate cancer, and each case included at least one tumor-positive core according to the pathology report. All sections from each block were analyzed without excluding any based on tumor fraction. This study was conducted in accordance with the Declaration of Helsinki and approved by the Northwestern University Institutional Review Board (protocol STU00213676) and conducted with a waiver of consent due to the retrospective nature of the study.

### Therapeutic outcomes

The second cohort was used to assess the predictive performance of the model for response to hormonal therapy. In this cohort, we focused on a single prognostic outcome: *hormonal therapy responsiveness*. HT responsiveness was defined as either a ≥90% reduction in prostate-specific antigen (PSA) levels from baseline prior to treatment or achievement of a PSA nadir below 0.2 ng/mL following initiation of therapy^33–35^. These criteria were selected based on their clinical relevance, as they reflect a robust early response to androgen deprivation and have been associated with favorable long-term outcomes. To ensure consistency in treatment exposure and data quality for analysis, we excluded patients whose prostate biopsy was performed three or more years prior to hormonal therapy initiation, as well as those with incomplete clinical or treatment information. Additionally, patients who received HT for two months or less were excluded to allow sufficient time for the therapy to exert a measurable biological effect on PSA. PSA measurements were assessed within a 3–6 month interval following therapy initiation to capture this early response. Patients who underwent triplet therapy (HT and chemotherapy) were excluded to avoid confounding effects from combination treatments that could influence the response independently of HT alone. Patients with baseline PSA levels below 2 ng/mL prior to hormonal therapy were also excluded, as such low values may reflect minimal disease burden and limit the ability to assess meaningful PSA-based treatment responses.

The third cohort focused on pathologic outcomes among patients who transitioned from active surveillance to definitive treatment with radical prostatectomy (associated with changes in cancer grade or PSA metrics in the majority of patients, 97.3%). The main outcome in this cohort was the presence of *adverse pathologic features (APFs)*, defined according to the National Comprehensive Cancer Network (NCCN) as any of the following findings on the prostatectomy specimen: Gleason Grade Group (GG) ≥ 3, seminal vesicle invasion (pT3b), or lymph node involvement (pN1). These pathologic features are considered indicators of aggressive disease and are associated with increased risk of recurrence and worse clinical prognosis. Supplementary Fig. 5A, B also provide a summary of the patient selection strategy for the second and third cohorts. Therapeutic outcomes were defined at the patient level, and predictions from all available slides were aggregated to derive a single patient-level measure, as described in the supplementary information.

### PAM50 and PSC prediction models

**Fig. 6:**
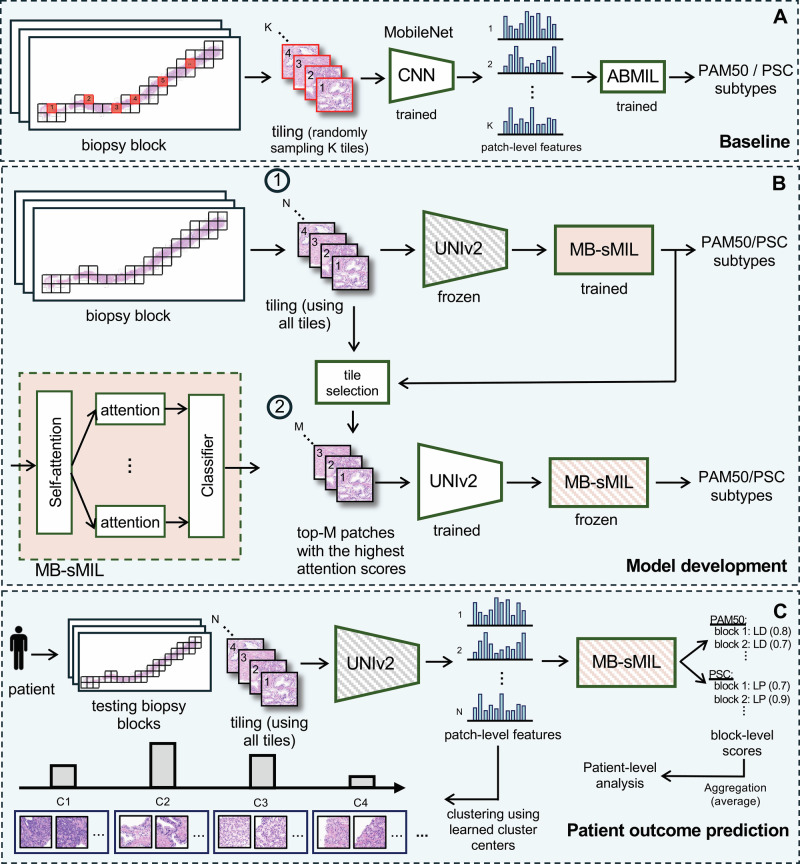
Training pipelines for PAM50 and PSC subtyping. **A** Baseline approach using a non-pathology vision CNN network coupled with an ABMIL model, trained end-to-end on randomly selected K image tiles. **B** Our novel approach uses UNIv2 finetuned through our proposed MB-sMIL (multi-branch self-attention multiple-instance learning) framework that captures morphological dependencies between image tiles. **C** Extraction of patient-level features using the trained UNIv2 and MB-sMIL models for therapeutic outcome predictions and clustering of patch-level morphologies to explore pathological associations with PAM50 and PSC subtypes.

We developed two approaches for image-based PAM50 and PSC subtyping using digital H&E-stained biopsy slides of prostate cancer. Our baseline model follows a typical architecture commonly used in digital pathology for image-level classification, integrating a non-pathology vision Convolutional Neural Network (CNN) with an attention-based multiple-instance learning (ABMIL) model^36^, trained end-to-end using a randomly sampled subset of K image tiles from each whole slide image. As our primary contribution, we developed a novel multiple-instance learning framework that finetunes UNIv2^37^, a pathology-trained foundational model, for image-based PAM50 and PSC subtyping using digital H&E-stained biopsy slides of prostate cancer. While pathology-trained foundational models like UNIv2 demonstrated superior generalizability compared to standard vision networks, they require task-specific fine-tuning to effectively adapt patch-level representations for optimal performance on specialized downstream tasks. Our targeted finetuning approach effectively bridges the gap between general patch-wise pathology representation and subtype-specific slide-level classification requirements. Furthermore, we propose the MB-sMIL model, a multi-branch MIL architecture that incorporates self-attention mechanisms to comprehensively capture inter-tile relationships within whole slide images. This approach is particularly beneficial for learning the morphological dependencies that inform molecular subtyping. Figure 6 illustrates our training pipeline for both methodologies. For molecular subtype prediction, our method analyzes the entire biopsy block rather than a single slide. This approach reflects the clinical reality that molecular subtypes are derived from the full biopsy specimen. We implemented two distinct computational approaches. Our baseline methodology employed a standard computational pathology pipeline wherein biopsy slides were divided into 448 × 448-pixel tiles, with K tiles randomly sampled from each biopsy block. These tiles were processed through an end-to-end trainable architecture comprising a CNN with MobileNetV2 backbone for feature extraction, followed by an attention-based multiple-instance learning (ABMIL) model for aggregating patch-level features into comprehensive block-level representations (Fig. 6A). The classification layer consisted of a fully connected layer with softmax activation, producing outputs corresponding to three PAM50 subtypes or four PSC subtypes. While this architecture enabled simultaneous training of both feature extraction and feature aggregation components, computational constraints limited processing to only K randomly selected tiles per slide. This approach, while it enables end-to-end training, risks missing important diagnostic regions that may not be captured in the sampled tiles.

Our main contribution extended beyond this conventional approach through a more comprehensive computational framework. While maintaining the same 448 × 448-pixel tiling strategy, our advanced method utilized all available tiles during model training, thereby maximizing exposure to morphological diversity within each specimen. The architecture integrated UNIv2, a pathology-trained foundational model, as the feature extraction component coupled with our novel multi-branch self-attention multiple-instance learning (MB-sMIL) framework for sophisticated feature aggregation (Fig. 6B). The training protocol proceeded through two sequential steps: initially, we preserved the pre-trained UNIv2 parameters while exclusively training the MB-sMIL component using all the tiles extracted from a biopsy block. The MB-sMIL architecture comprised a self-attention layer with 16 heads and embedding dimension of 256 to learn dependencies between tiles, capturing contextual relationships across tissue regions. The resulting transformed features were then passed through multi-branch pathways (one branch for each class) with gated attention layers utilizing sigmoid and tanh activations to create class-specific attention weights and feature representations. These class-specific representations were subsequently processed through a dense layer with softmax activation to predict the final subtype probabilities. Subsequently, in the second step, we conducted targeted fine-tuning of the UNIv2 model while maintaining fixed MB-sMIL parameters, utilizing only the M most informative tiles as determined by attention weights from the first phase. This complementary approach facilitated fine-tuning of the large-scale UNIv2 foundational model on diagnostically relevant tissue regions for a better molecular subtype classification.

To investigate the relationship between model-predicted molecular subtypes and clinical outcomes, including hormone therapy response and adverse pathologic features, we applied a confidence threshold to the predicted scores. The predicted molecular subtype scores below 0.5 were set to zero, while those equal to or above this value were retained to reduce the impact of low-confidence predictions. For cases with multiple blocks per biopsy, retained scores were first averaged at the core and then at the block level to generate patient-level scores, which were analyzed for associations with the outcomes (Fig. 6C).

### Statistical analysis

Categorical clinical variables were compared among the PAM 50 subtypes and the PSC subtypes. Additionally, the distribution of clinical variables was also compared by hormonal therapy responsiveness and adverse pathologic features. Continuous variables were summarized as median and interquartile ranges (IQR) and compared between the groups using the Wilcoxon rank test, ANOVA, and the Kruskal-Wallis test, as appropriate. Categorical variables were summarized as counts and frequencies and compared between the groups using ${\chi }^{2}$ and Fisher’s exact tests. The Point-biserial correlation coefficients were calculated to assess the relationship between predicted molecular subtype scores and the outcomes. Univariable logistic regression analysis was also performed to determine the factors associated with hormonal therapy responsiveness and adverse pathologic features, with scores evaluated per 0.1-unit increase. Receiver operating characteristic (ROC) curve analysis was performed to evaluate the discriminative ability of the model-identified pathological features. The area under the ROC curve (AUC) and F1 score were calculated to quantify the subtype prediction performance, with a bootstrapping technique using 1000 iterations employed to assess the variability and generate 95% confidence intervals. For semantic features generated by our models, a dimension reduction technique known as Uniform Manifold Approximation and Projection (UMAP) was employed to visualize feature distributions across PAM50 and PSC subtypes. For all analyses, a two-sided *p*-value < 0.05 was considered statistically significant.

## Supplementary information

Supplementary Information_clean_012626

## Data Availability

The datasets analyzed in the current study are not publicly available as they consist of electronic health records, and institutional policies require a data-sharing agreement for access.

## Code availability

Source code is available via GitHub at https://github.com/RaminNateghi/PCaSub-MB-sMIL.
