# Supplementary material for: Prediction of molecular subtypes from histology: AI-driven analysis of prostate cancer morphological patterns and therapeutic implications
Source: NPJ Precis Oncol. 2026 Mar 19;10:225. doi: 10.1038/s41698-026-01335-y (PMC13270054; doi:10.1038/s41698-026-01335-y)
Supplement: Supplementary file 1 — Supplementary Information_clean_012626 [file 41698_2026_1335_MOESM1_ESM.pdf]

Supplementary Information

Supplementary Table 1: Demographic and clinical characteristics with respect to PAM50 subtypes in the first cohort

	Overall N = 431[1]	PAM50 – Luminal A (LA) N = 194[1]	PAM50 – Luminal B (LB) N = 157[1]	PAM50 – Basal (B) N = 80[1]	p-value[2]
Age, median (IQR)	68 (61.6 – 74)	66.5 (59 – 72)	69 (63 – 75)	67 (63 – 73)	**< 0.001**
Has family history, n (%)	107 (24.83%)	46 (23.12%)	39 (24.07%)	22 (25.88%)	0.62
Race, n (%)					**0.04**
Black	64 (14.85%)	37 (18.59%)	19 (11.73%)	8 (9.41%)	
White	289 (66.28%)	123 (61.81%)	103 (63.58%)	63 (74.12%)	
Other / Declined	78 (18.1%)	35 (18.04%)	34 (21.66%)	9 (11.25%)	
BMI, median (IQR)	27.62 (24.97 - 30.89)	27.9 (25.2 – 31.9)	27.5 (24.7 - 30)	26.9 (24.9 – 29.8)	0.59
CCI, median (IQR)	5 (4 – 7)	5 (4 - 7)	6 (4 – 8)	5 (4 - 7.25)	**0.02**
GG at diagnosis, n (%)					**< 0.001**
1	52 (12.06%)	31 (15.58%)	12 (7.41%)	9 (10.59%)	
2	276 (64.04%)	131 (65.83%)	87 (53.7%)	58 (68.24%)	
3	80 (18.56%)	24 (12.06%)	43 (26.54%)	13 (15.29%)	
4	15 (3.48%)	6 (3.02%)	9 (5.56%)	0 (0%)	
5	8 (1.86%)	2 (1.01%)	6 (3.7%)	0 (0%)	
PSA at diagnosis, median (IQR)	6.07 (3.67 – 9.08)	5.13 (3.78 - 6.96)	5.32 (4.07 - 8.00)	5.44 (3.65 - 7.44)	0.3
Had mpMRI, n (%)					0.64
Yes	407 (94.43%)	181 (90.95%)	150 (92.59%)	76 (89.41%)	
No	24 (5.57%)	13	7	4	
PIRADS, if available, n (%)					**0.03**
0	9 (2.09%)	5 (2.51%)	2 (1.23%)	2 (2.35%)	
1	1 (0.23%)	0 (0%)	0 (0%)	1 (1.18%)	
2	2 (0.46%)	2 (1.01%)	0 (0%)	0 (0%)	
3	46 (10.67%)	23 (11.56%)	13 (8.02%)	10 (11.76%)	
4	251 (58.24%)	117 (58.79%)	84 (51.85%)	50 (58.82%)	
5	92 (21.35%)	32 (16.08%)	48 (29.63%)	12 (14.12%)	
NA	30 (6.96%)				
Prostate volume, if available, median (IQR)	42 (29 – 56)	42.9 (32.8 – 57)	40 (26.3 – 53)	45 (29 - 61.1)	0.11
Maximum GP4%, median (IQR)	10 (5 – 30)	10 (5 – 30)	15 (10 – 60)	10 (5 – 22.5)	**0.005**
Maximum core-level cancer %, median (IQR)	45 (20 – 70)	40 (20 – 60)	60 (35 – 80)	20 (10 – 50)	**<0.001**
PSAD, if available, median (IQR)	0.13 (0.08 – 0.19)	0.118 (0.08 - 0.169)	0.15 (0.093 - 0.22)	0.137 (0.082 - 0.188)	0.41

[1]Median (Q1, Q3); n (%) [2]Wilcoxon rank sum test; Pearson's Chi-squared test; Fisher's exact test

Supplementary Table 2: Demographic and clinical characteristics with respect to PSC subtypes in the first cohort

	Overall N = 431[1]	PSC - Luminal Differentiated (LD) N = 139[1]	PSC - Luminal Proliferating (LP) N = 95[1]	PSC - Basal Immune (BI) N = 188[1]	PSC - Basal Neuroendocrine (BN) N = 9[1]	p-value[2]
Age, median (IQR)	68 (61.6 – 74)	67 (60.5 – 72)	69 (63 - 75.5)	67 (61 – 74)	71 (67 – 72)	0.08
Has family history, n (%)	107 (24.83%)	33 (22.92%)	26 (26%)	45 (23.32%)	3 (21.43%)	0.75
Race, n (%)						0.14
Black	64 (14.85%)	18 (12.5%)	20 (20%)	26 (13.47%)	0 (0%)	
White	289 (66.28%)	90 (62.5%)	60 (60%)	130 (67.36%)	9 (64.29%)	
Other / Declined	78 (18.1%)	31 (22.3%)	15 (15.79%)	32 (17.02%)	0 (0%)	
BMI, median (IQR)	27.62 (24.97 - 30.89)	26.9 (24.4 -29.8)	28.2 (25.5 - 32.5)	27.7 (25.3 - 30.8)	28.6 (24.9 - 29.4)	0.14
CCI, median (IQR)	5 (4 – 7)	5 (4 - 7)	6 (5 - 8)	5 (4 - 7)	9 (6 – 11)	**0.003**
GG at diagnosis, n (%)						**< 0.001**
1	52 (12.06%)	22 (15.28%)	4 (4%)	24 (12.44%)	2 (14.29%)	
2	276 (64.04%)	102 (70.83%)	56 (56%)	112 (58.03%)	6 (42.86%)	
3	80 (18.56%)	14 (9.72%)	24 (24%)	41 (21.24%)	1 (7.14%)	
4	15 (3.48%)	0 (0%)	9 (9%)	6 (3.11%)	0 (0%)	
5	8 (1.86%)	1 (0.69%)	2 (2%)	5 (2.59%)	0 (0%)	
PSA at diagnosis, median (IQR)	6.07 (3.67 – 9.08)	5.29 (3.85 - 6.91)	4.92 (3.80 - 6.51)	5.62 (4.01 - 8.08)	4.57 (2.41 - 7.34)	0.35
Had mpMRI, n (%)						**0.03**
Yes	407 (94.43%)	135 (93.75%)	93 (93%)	170 (88.08%)	9 (64.29%)	
No	24 (5.57%)	4	2	18	0	
PIRADS, if available, n (%)						0.26
0	9 (2.09%)	4 (2.78%)	2 (2%)	3 (1.55%)	0 (0%)	
1	1 (0.23%)	0 (0%)	0 (0%)	1 (0.52%)	0 (0%)	
2	2 (0.46%)	0 (0%)	0 (0%)	2 (1.04%)	0 (0%)	
3	46 (10.67%)	19 (13.19%)	5 (5%)	22 (11.4%)	0 (0%)	
4	251 (58.24%)	87 (60.42%)	59 (59%)	99 (51.3%)	6 (42.86%)	
5	92 (21.35%)	21 (14.58%)	26 (26%)	42 (21.76%)	3 (21.43%)	
NA	30 (6.96%)					
Prostate volume, if available, median (IQR)	42 (29 – 56)	42.3 (29.9 -51.7)	42.4 (27.8 -55.4)	41 (31.4 - 62)	26.3 (21 - 56.6)	0.31
Maximum GP4%, median (IQR)	10 (5 – 30)	10 (5 – 20)	20 (7.5 – 45)	10 (5 – 40)	10 (5 – 30)	0.094
Maximum core-level cancer%, median (IQR)	45 (20 – 70)	40 (20 – 65)	50 (20 – 80)	40 (15 – 75)	50 (10 – 70)	0.11
PSAD, if available, median (IQR)	0.13 (0.08 – 0.19)	0.12 (0.0875 -0.181)	0.13 (0.08 -0.19)	0.13 (0.09 -0.19)	0.17 (0.07 - 0.206)	0.92

[1]Median (Q1, Q3); n (%) [2]Wilcoxon rank sum test; Pearson's Chi-squared test; Fisher's exact test

Supplementary Table 3: Demographic and clinical characteristics with respect to HT responsiveness for the second cohort

	Overall<br>N = 131[1]	HT Responsive<br>N = 112[1]	HT non-responsive<br>N = 19[1]	p-value[2]
Age, median (IQR)	75.00 (68.00, 79.00)	73.00 (68.00, 79.00)	75.00 (68.00, 79.00)	0.7
Has family history, n (%)				0.7
0	91 (69%)	77 (69%)	14 (74%)	
1	40 (31%)	35 (31%)	5 (26%)	
Race, n (%)				>0.9
White	87 (66%)	75 (67%)	12 (63%)	
Black	30 (23%)	25 (22%)	5 (26%)	
Other/Declined	14 (11%)	12 (11%)	2 (11%)	
BMI, median (IQR)	28.98 (25.76, 31.74)	29.11 (25.78, 32.03)	27.91 (24.66, 31.19)	0.4
CCI, median (IQR)	8.00 (6.00, 11.00)	8.00 (6.00, 11.00)	6.00 (5.00, 9.00)	0.2
ADT Type, n (%)				0.018
Single	39 (30%)	29 (26%)	10 (53%)	
Double	92 (70%)	83 (74%)	9 (47%)	
GG at biopsy prior to HT, n (%)				**0.020**
2	24 (19%)	17 (15%)	7 (37%)	
3	39 (30%)	32 (29%)	7 (37%)	
4	18 (14%)	15 (14%)	3 (16%)	
5	48 (37%)	46 (42%)	2 (11%)	
Unknown	2	2	0	
ADT Clin Stage Group, n (%)				0.7
Stage I	9 (30%)	7 (28%)	2 (40%)	
Stage IIC	1 (3.3%)	1 (4.0%)	0 (0%)	
Stage IIIC	20 (67%)	17 (68%)	3 (60%)	
Unknown	101	87	14	
M Stage, n (%)				0.5
M0	17 (81%)	12 (75%)	5 (100%)	
M1b	4 (19%)	4 (25%)	0 (0%)	
Unknown	110	96	14	
N Stage, n (%)				0.6
cN0	16 (76%)	11 (69%)	5 (100%)	
cN1	4 (19%)	4 (25%)	0 (0%)	
cNX	1 (4.8%)	1 (6.3%)	0 (0%)	
Unknown	110	96	14	
T Stage, n (%)				0.5
Other	1 (4.8%)	1 (6.3%)	0 (0%)	
T1	10 (48%)	6 (38%)	4 (80%)	
T2	5 (24%)	5 (31%)	0 (0%)	
T3	4 (19%)	3 (19%)	1 (20%)	
T4	1 (4.8%)	1 (6.3%)	0 (0%)	
Unknown	110	96	14	
Prior Biopsy Fraction of Positive Cores, median (IQR)	0.50 (0.33, 0.71)	0.55 (0.33, 0.75)	0.33 (0.13, 0.54)	**0.003**
PSA prior to hormonal therapy, median (IQR)	10.21 (5.61, 23.26)	10.61 (5.01, 24.72)	9.43 (6.89, 16.60)	>0.9
Prior Biopsy, Maximum GP4%, median (IQR)	60 (0 – 80)	40 (10 – 75)	60 (0 – 80)	0.84
Prior Biopsy, Maximum core-level cancer%, median (IQR)	90.00<br>(75.00, 100.00)	90.00<br>(80.00, 100.00)	80.00<br>(40.00, 90.00)	**0.005**
Unknown	1	1	0	

[1]Median (Q1, Q3); n (%) [2]ANOVA and Kruskal Wallis test; Pearson's Chi-squared test; Fisher's exact test

Supplementary Table 4: Demographic and clinical characteristics with respect to APFs for the third cohort

	Overall N = 122[1]	APFs N = 39[1]	No APFs N = 83[1]	p-value[2]
Age, median (IQR)	68 (62 - 72)	68 (63 - 70)	68 (62 - 74)	0.7
Family History, n (%)				0.2
0	90 (74%)	26 (67%)	64 (77%)	
1	32 (26%)	13 (33%)	19 (23%)	
BMI, median (IQR)	27.79 (24.11, 29.84)	27.31 (23.71, 28.47)	28.37 (24.52, 29.99)	**0.06**
Race, n (%)				0.3
White	86 (70%)	24 (62%)	62 (75%)	
Black	16 (13%)	7 (18%)	9 (11%)	
Other/Declined	20 (17%)	8 (21%)	12 (14%)	
Biopsy Prior to RP, Gleason Grade, n (%)				**<0.001**
1	10 (8.2%)	2 (5.1%)	8 (9.6%)	
2	74 (61%)	13 (33%)	61 (73%)	
3	27 (22%)	16 (41%)	11 (13%)	
4	8 (6.6%)	5 (13%)	3 (3.6%)	
5	3 (2.5%)	3 (7.7%)	0 (0%)	
PIRADS n (%)				0.4
2	3 (4.4%)	0 (0%)	3 (6.0%)	
3	13 (19%)	2 (11%)	11 (22%)	
4	46 (68%)	13 (72%)	33 (66%)	
5	6 (8.8%)	3 (17%)	3 (6.0%)	
Biopsy Prior to RP, Maximum GP4%, median (IQR)	20 (10 - 50)	55 (10 - 70)	10 (10 – 30)	**0.008**
Biopsy Prior to RP, Maximum core-level cancer%, median (IQR)	50 (5 - 80)	50 (5 - 80)	30 (5 – 70)	0.2
Time on active surveillance , median (IQR)	558.50 (409.00, 1,098.00)	624.00 (409.00, 1,174.00)	547.00 (409.00, 1,065.00)	0.7

[1]Median (Q1, Q3); n (%) [2]ANOVA and Kruskal Wallis test; Pearson's Chi-squared test; Fisher's exact test

Supplementary Table 5: Association between predicted molecular subtype scores and HT outcomes using an alternative confidence threshold of 0.3

Model scores	HT non-responsive N = 19[1]	HT Responsive N = 112[1]	Overall N = 131[1]	p-value[2]	Correlation[3]	p-value[3]
Luminal A	0.49 (0.37, 0.66)	0.37 (0.16, 0.57)	0.39 (0.22, 0.60)	0.39	-0.18	0.38
Luminal B	0.08 (0.00, 0.17)	0.22 (0.08, 0.42)	0.18 (0.06, 0.39)	**0.002**	**0.2447**	**0.005**
Basal	0.00 (0.00, 0.06)	0.00 (0.00, 0.00)	0.00 (0.00, 0.00)	0.24	-0.0960	0.2751
Luminal differentiated	0.33 (0.25, 0.49)	0.31 (0.16, 0.47)	0.32 (0.18, 0.48)	0.61	-0.0267	0.7616
Luminal proliferating	0.07 (0.01, 0.13)	0.10 (0.05, 0.19)	0.11 (0.04, 0.18)	**0.036**	0.1439	0.1008
Basal immune	0.62 (0.44, 0.74)	0.51 (0.39, 0.66)	0.53 (0.43, 0.69)	0.32	-0.1023	0.3125
Basal neuroendocrine	0.04 (0.01, 0.05)	0.04 (0.02, 0.05)	0.03 (0.02, 0.06)	0.608	0.0366	0.6774

[1]Median (Q1, Q3); n (%) [2]Wilcoxon rank sum test, Point biserial[3]

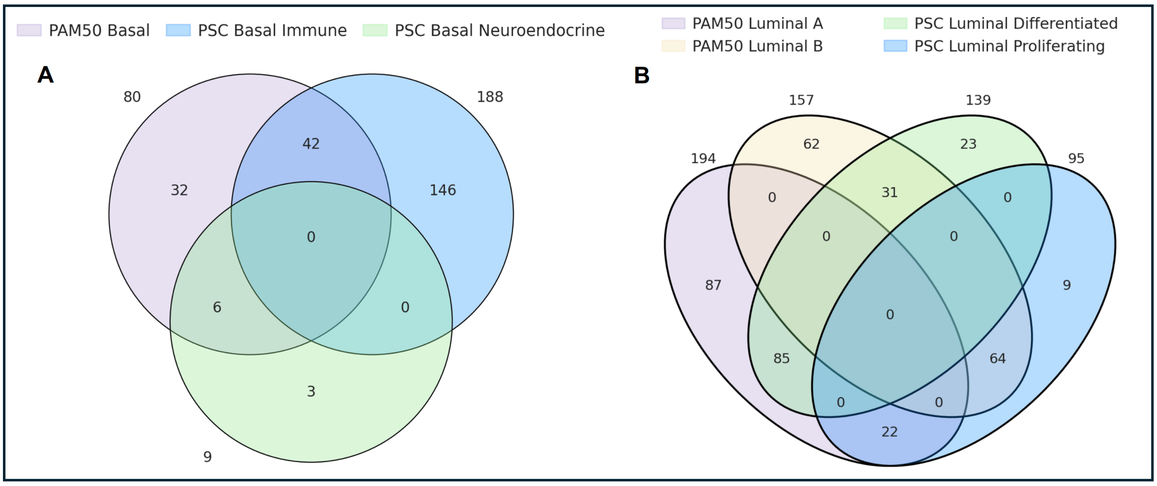

Supplementary Figure 1. Distribution of biopsy blocks in the first cohort across PAM50 and PSC subtypes: Basal (A) and luminal (B) subtypes.

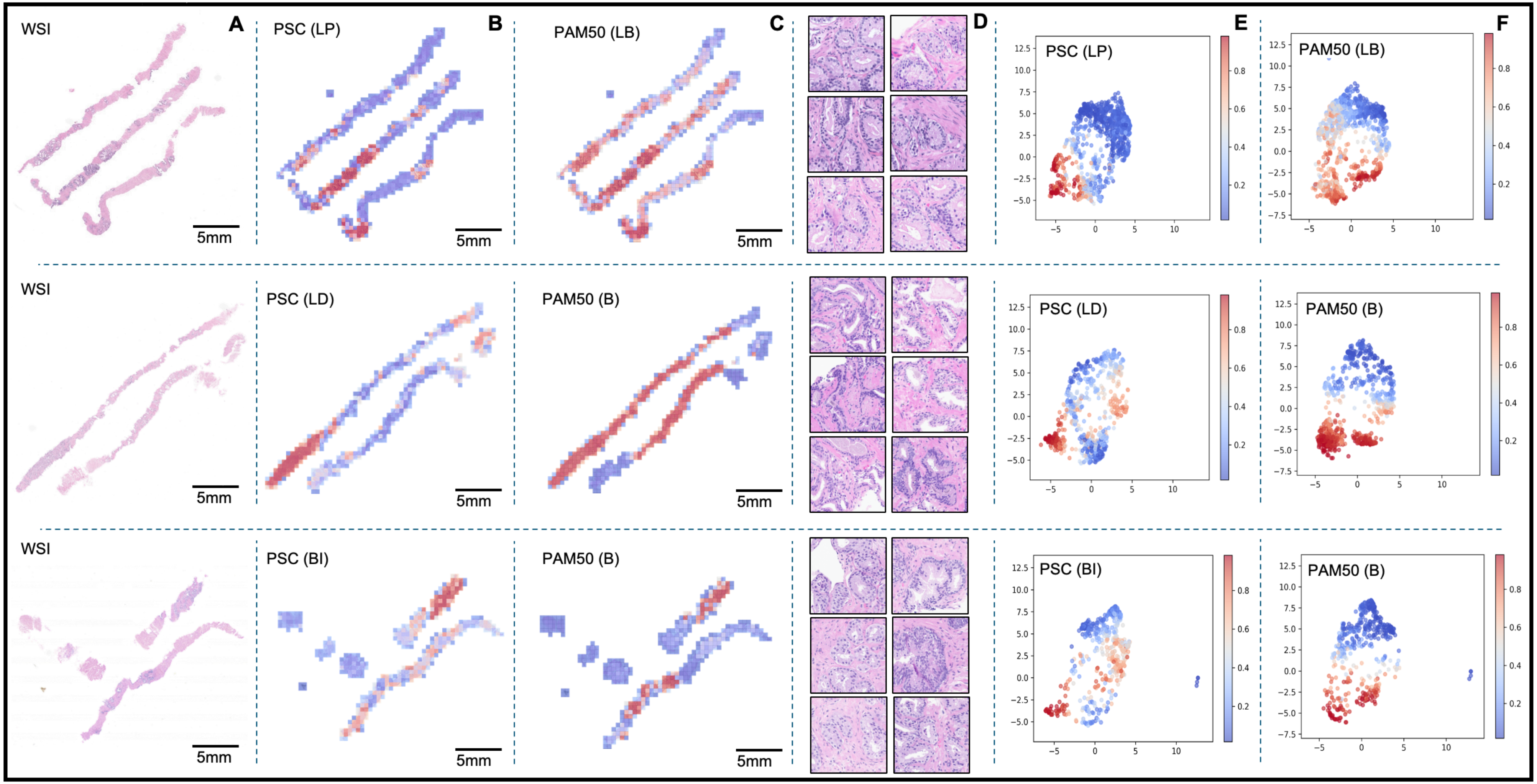

Supplementary Figure 2. Visualizations of attention maps and feature embeddings for additional testing slides. (A) Whole-slide images of representative biopsy samples. (B-C) Attention maps overlaid on H&E slides, highlighting regions important for PSC and PAM50 subtyping, respectively. (D) Representative tiles ranked by attention weights for PAM50 (first column) and PSC (second column) subtyping. (E-F) UMAP embeddings of tile features for the PSC and PAM50 models, color-coded by attention weights (red indicates higher attention, blue lower attention).

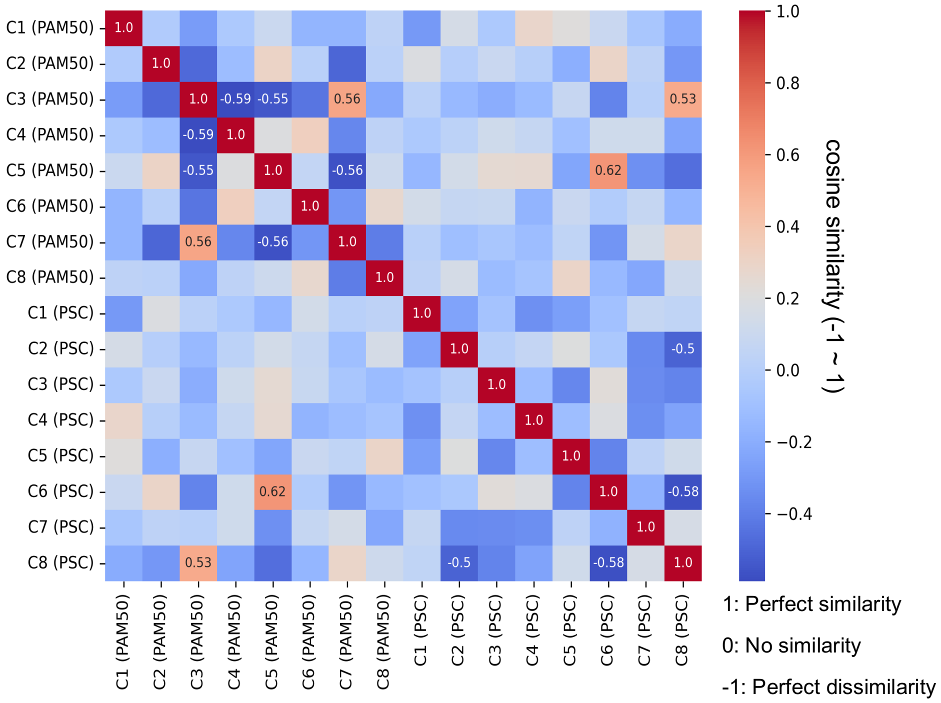

Supplementary Figure 3. Correlation matrix between clusters identified by the PAM50 and PSC subtyping models. The heatmap displays cosine similarities ranging from -1 (perfect dissimilarity, blue) to +1 (perfect similarity correlation, red), with values closer to 0 indicating no similarity. For clarity, only correlation coefficients with absolute values greater than ±0.5 are labeled. Overall, there is no significant correlation between clusters from the two models, indicating they capture distinct pathological patterns. Only C5 (PAM50) shows mild correlations with C6 (PAM50), suggesting overlap in these specific cluster features.

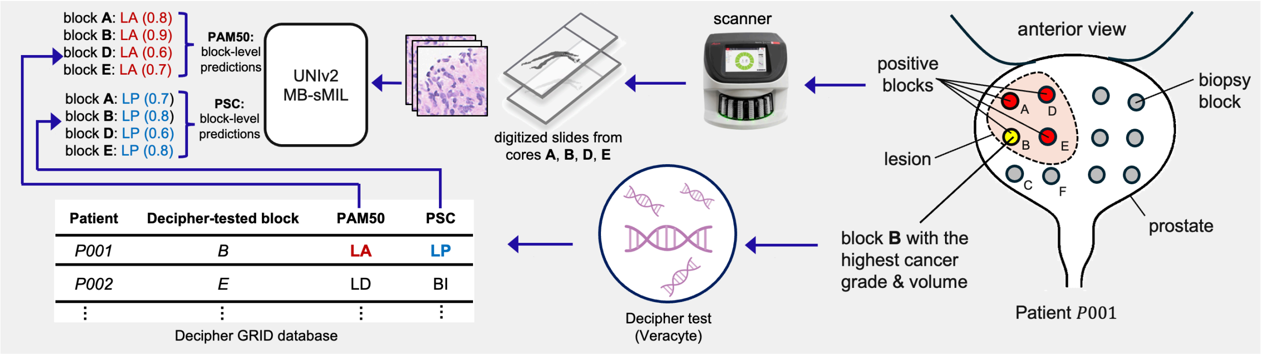

Supplementary Figure 4. Workflow for mapping Decipher-derived block-level molecular subtypes to corresponding digitized pathology slides. This figure illustrates the workflow for integrating genomic molecular subtypes with pathology data. For each patient, the biopsy block with the highest tumor grade and volume was selected for Decipher testing to generate PAM50 and PSC subtypes. For our analysis, all tumor-containing blocks from the same biopsy and patient were assigned the corresponding Decipher-derived subtypes, which served as ground truth in our digital pathology pipeline.

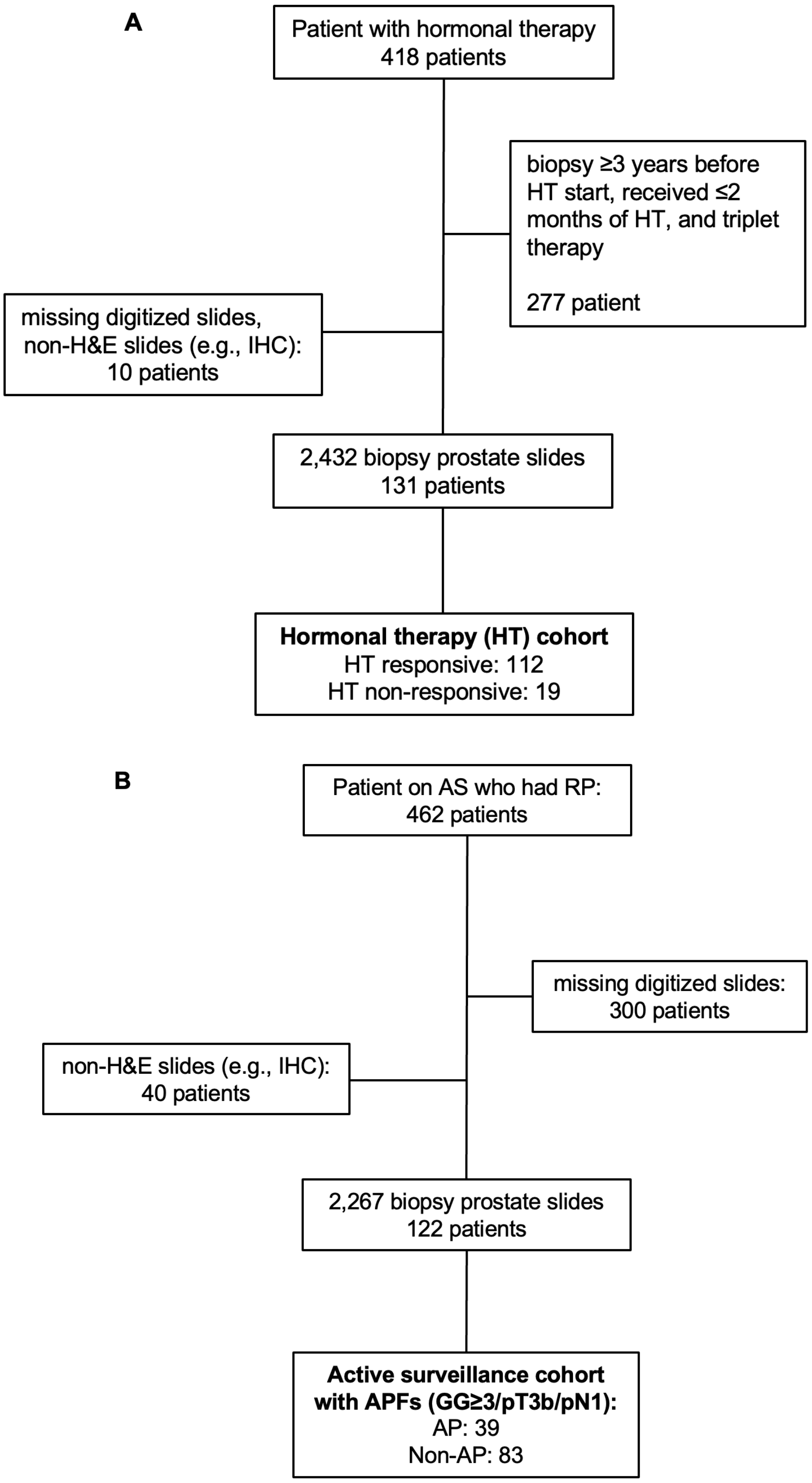

Supplementary Figure 5. Cohort selection for analyses of hormonal therapy (HT) responsiveness and adverse pathologic features (APFs) following active surveillance. (A) Flowchart illustrating inclusion and exclusion criteria for the cohort used to evaluate HT responsiveness. (B) Flowchart depicting cohort selection for patients transitioning from active surveillance to radical prostatectomy, used to assess the prediction of APFs, defined as GG ≥3, pT3b, or pN1 disease.
